# An efficient grasping shared control architecture for unpredictable and unspecified tasks

**DOI:** 10.3389/fnbot.2024.1429952

**Published:** 2024-09-11

**Authors:** Shaowen Cheng, Yongbin Jin, Yanhong Liang, Lei Jiang, Hongtao Wang

**Affiliations:** ^1^Center for X-Mechanics, Zhejiang University, Hangzhou, China; ^2^ZJU-Hangzhou Global Scientific and Technological Innovation Center, Zhejiang University, Hangzhou, China; ^3^State Key Laboratory of Fluid Power and Mechatronic System, Zhejiang University, Hangzhou, China; ^4^Institute of Applied Mechanics, Zhejiang University, Hangzhou, China

**Keywords:** human-centered robotics, shared control, teleoperation, grasping, motion planning

## Abstract

Robot control in complex and unpredictable scenarios presents challenges such as adaptability, robustness, and human-robot interaction. These scenarios often require robots to perform tasks that involve unknown objects in unstructured environments with high levels of uncertainty. Traditional control methods, such as automatic control, may not be suitable due to their limited adaptability and reliance on prior knowledge. Human-in-the-loop method faces issues such as insufficient feedback, increased failure rates due to noise and delays, and lack of operator immersion, preventing the achievement of human-level performance. This study proposed a shared control framework to achieve a trade-off between efficiency and adaptability by combing the advantages of both teleoperation and automatic control method. The proposed approach combines the advantages of both human and automatic control methods to achieve a balance between performance and adaptability. We developed a linear model to compare three control methods and analyzed the impact of position noise and communication delays on performance. The real-world implementation of the shared control system demonstrates its effectiveness in object grasping and manipulation tasks. The results suggest that shared control can significantly improve grasping efficiency while maintaining adaptability in task execution for practical robotics applications.

## 1 Introduction

Research on robot control has been an active area of investigation for several decades, with applications in a wide range of fields including hazardous environments (Tsitsimpelis et al., 2019), disaster relief (Norton et al., 2017), deep space exploration (Diftler et al., 2012), and deep sea exploration (Khatib et al., 2016). With the advent of deep learning techniques, automatic control methods have undergone significant advancements in recent years. These methods often excel in controlling efficiency for a single purpose and within a specific scenario, achieving high efficiency of manipulation. However, they lack task adaptability, particularly when it comes to achieving specific grasps for the same object based on different usage intentions (Brahmbhatt et al., 2019). The integration of human intelligence enables robots to increase the variety of objects used and effectively deal with unpredictable problems in unstructured environments (Hokayem and Spong, 2006). Therefore, the use of human intelligence in teleoperation methods has also received extensive research attention (Moniruzzaman et al., 2022). Both of these methods have their respective advantages in terms of task efficiency and adaptability.

**Figure 1:**
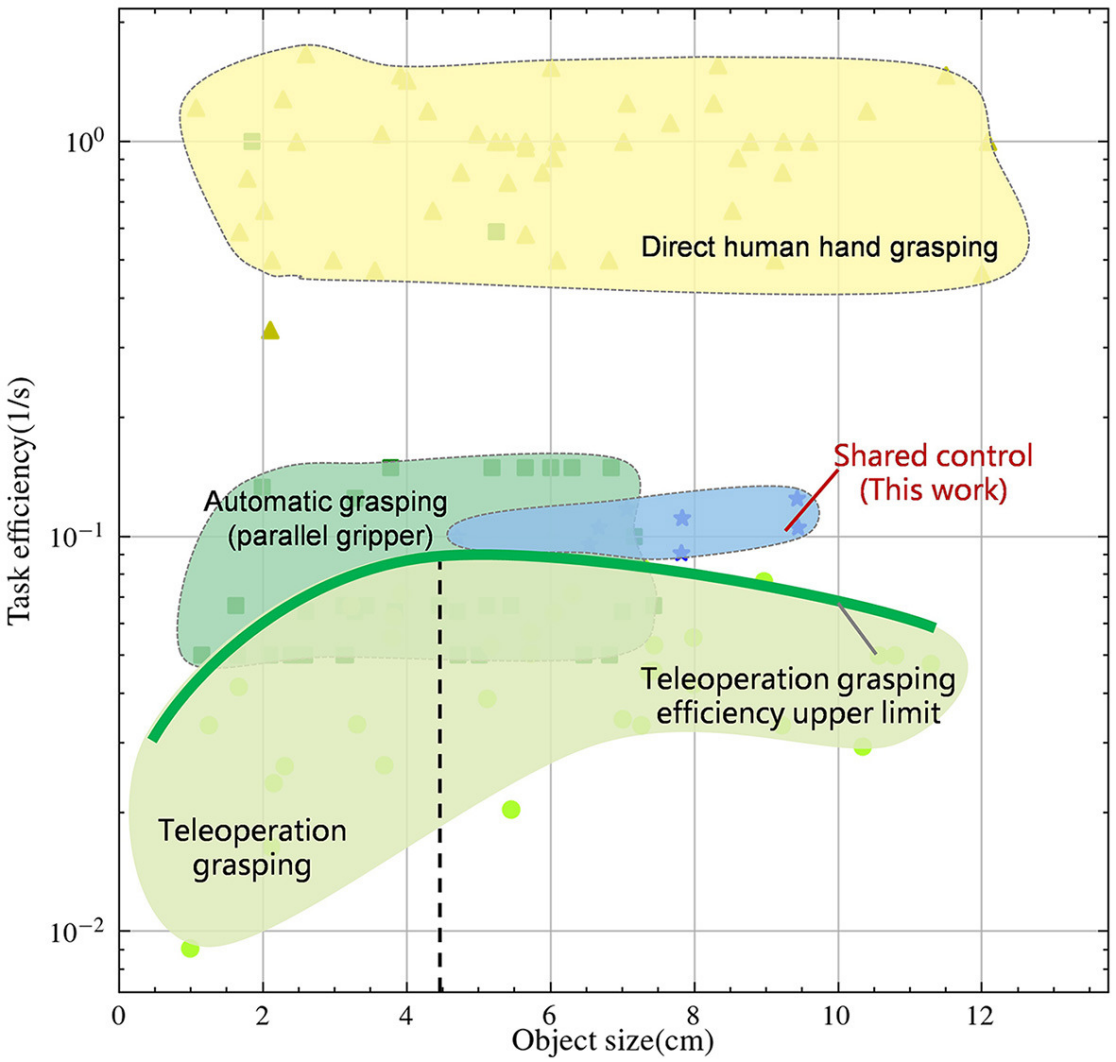
Task efficiency-Object size relationship between direct human control, auto control and teleoperation in logarithmic scales. Detailed data are in the Supplementary material.

In Figure 1, we investigated the efficiency and adaptability of different control methods and compared them with human performance, all depicted on logarithmic scales. Reasonably, direct human hand grasping demonstrates the highest efficiency and adaptability. Despite modern automatic control algorithms achieving planning times under 1 second (Morrison et al., 2018), their overall work efficiency remains an order of magnitude lower than human operation, particularly noticeable with objects under 8 cm in size, limited by clamp size. Teleoperation can adapt to object sizes similarly to human, with peak efficiency occurring around medium-sized objects, approximately 4.5 cm in size. As objects deviate from this range in either direction, teleoperation efficiency declines, as indicated by the green bold curve.

Data-driven grasp synthesis are widely used in robotic grasping and manipulation (Mahler et al., 2017, 2019; Morrison et al., 2018). Grasping strategies using parallel clamps and suction cup grippers have been widely used. This approach often utilizes supervised learning to sample and rank candidate grasps. The synergy of precise object positioning through automatic control and the low-dimensional end effector enables rapid grasping. Nonetheless, as shown in Figure 1, this approach is limited by the size of end effector and cannot accommodate objects of all sizes. In recent years, there has been a widespread adoption of model-based approaches (Nagabandi et al., 2019) and model-free reinforcement learning techniques (Chen et al., 2021). These methods leverage deep learning techniques to facilitate the learning of dexterity in multi-fingered hands to enhance the adaptability of this method. However, these methods are known to face challenges due to the high dimensional search space, resulting in low success rates and robot hand configurations that do not emulate natural human movements. As an alternative, learning with demonstration (Rajeswaran et al., 2018) has shown promise as a method to reduce search space and increase the success rate. However, the quality of the demonstration data is often a bottleneck in this approach. Although current state-of-the-art automatic control algorithms have demonstrated success in solving specific tasks and adaptability to objects of various sizes, they still face challenges when it comes to handling unpredictable scenarios. Even with recent advances in deep learning, the challenge of achieving human-like dexterity and adaptability in robotic control remains an open research question, requiring further investigation.

**Figure 2:**
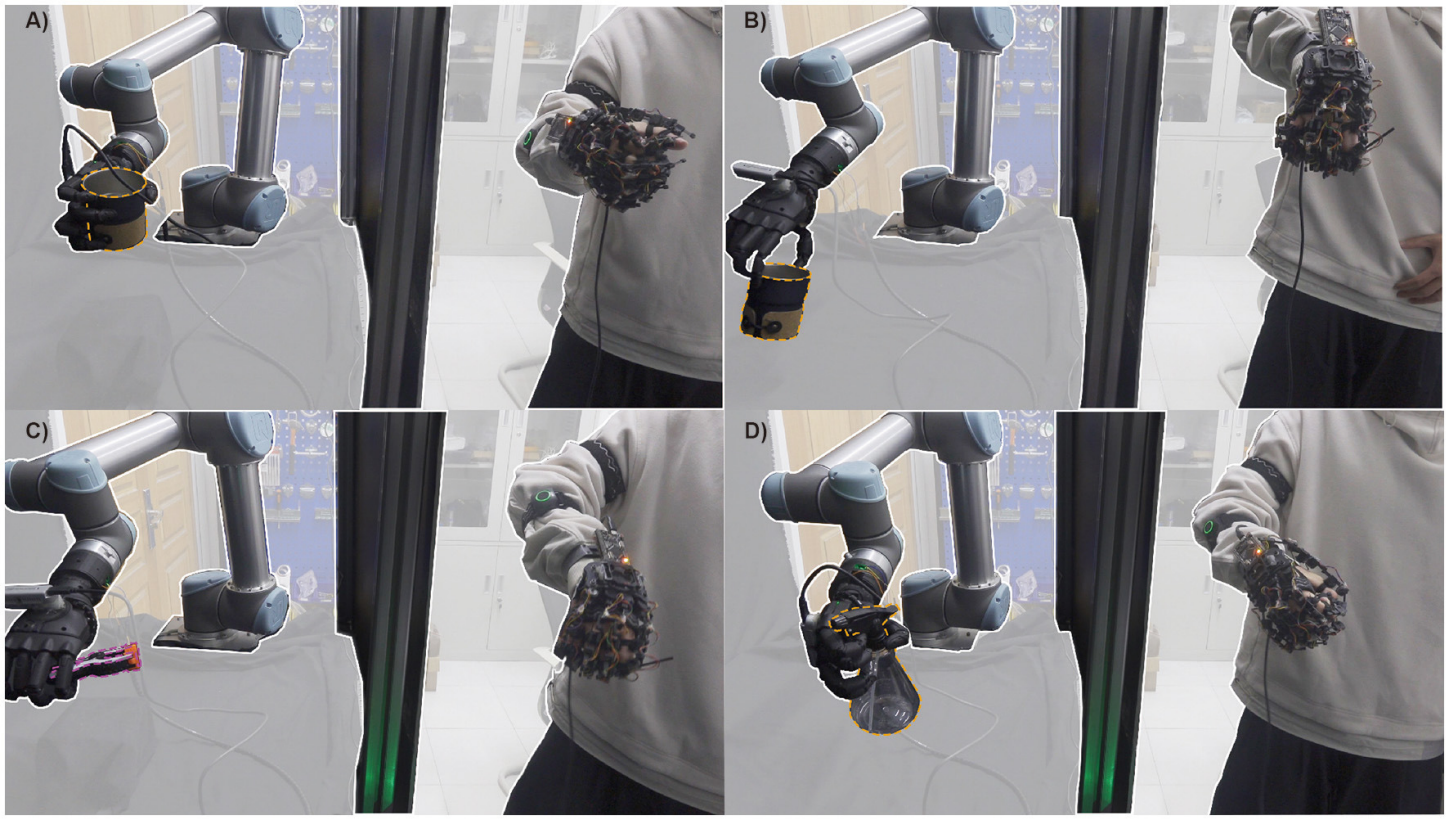
Shared control systems can accomplish different kinds of tasks, e.g., interacting with the cup for different functional intent: use **(A)** and handoff **(B)**; holding the clamp **(C)**; extrusion nozzle of windex bottle **(D)**.

Human-in-the-loop can adapt to complex scenarios with the help of cameras, monitors, motion capture system, etc. It has been shown to be a promising method for achieving high-level control of robots, particularly in tasks that require human-like dexterity and adaptability. As illustrated in Figure 2, due to the highly anthropomorphic structure of the end effector, which resembles the human hand, this approach achieves versatility in grasping objects of all sizes, making it a widely adopted method in robot grasping and manipulation. In addition, research has shown that contact location is largely dependent on post-action with objects (Brahmbhatt et al., 2019). However, teleoperation has certain limitations that need to be addressed. One of the principal hurdles stems from the inescapable sensor noise and communication channel delays (Farajiparvar et al., 2020), which may precipitate a surge in failure rates. Another concern pertains to the dearth of substantial feedback, potentially resulting in reduced operator immersion and an upsurge in time and labor requirements throughout the operation (Moniruzzaman et al., 2022). These limitations have caused teleoperation to operate significantly below human-level efficiency. Fishel et al. (2020) found the performance of the world's most advanced human operator piloting the telerobot to be anywhere from 4 to 12 times slower than that same operator with their bare hands. As such, the shared control method (Kim et al., 2006) has been proposed as a means of combining the advantages of both methods to achieve “fast and accurate” grasping and tool use.

For human, individuals do not possess exact knowledge of the object's position, but instead receive information about the relative position of the human hand and the object, and subsequently send high-frequency velocity commands to the hand to complete the task (Jeka et al., 2004). However, when the end effector changes from a human hand to an anthropomorphic hand, the change in mechanism and the increase in delay and noise make control challenging. This can lead to a prolonged operation time and a decreased success rate. In this study, we proposed a shared control framework for robotic grasping tasks. The proposed approach combines the advantages of both human and automatic control methods and aims to achieve a balance between performance and adaptability. This study proposes integrating a camera into the teleoperation system to infer the position and size of the object, thereby enabling automatic control to initialize the grasping position. The human operator then takes over control for the adaptability as the human control.

To demonstrate the effectiveness of this proposed approach, a model was constructed to compare the performance metrics of shared control, human control, and automatic control in the presence of noise. It is assumed that the velocity during the operation follows a linear relationship (Jerbi et al., 2007), where the speed of the motion is proportional to the distance from the target position. The modeling results indicate that the shared control method exhibits similar trajectory smoothness and speed as the automatic control method and similar grasping accuracy as the human control method, thus achieving a balance between performance and adaptability. Furthermore, the impact of delay and error on the system was analyzed. The results indicate that only large delays have a significant impact on the system due to human involvement.

This work presents a shared control framework that combines human intelligence and automatic control to improve the performance of robotic grasping tasks in unpredictable environments, as shown in Figure 2. Our main contributions include:

(1) This work proposes a human-robot shared control method by integrating human control with autonomous control. The simulation and practical deployment of this method demonstrates that it effectively resolves the conflict between efficiency and adaptability.
(2) A linear dynamic model was established to quantitatively analyze the performance of the three control strategies.
(3) The proposed quantitative analysis method has been further extended to analyze the impact of delays and position estimation errors on human-in-loop systems.

## 2 Related work

Research in the field of shared control and teleoperation with assistance aims to enable robots to help operators complete desired tasks more efficiently. In teleoperation with assistance, the system primarily helps to simplify and facilitate the user's manual input, ensuring the user retains direct control over the device. In contrast, shared control systems aim to understand and predict the user's intent, allowing the system to autonomously perform some tasks and reduce the cognitive load on the user.

Recent advancements in machine learning techniques have led to the development of methods such as glove-based (Wang and Popović, 2009) and vision-based (Handa et al., 2020) tracking to achieve accurate joint position tracking. The structural differences between human and robot hands have led to the proposal of techniques such as kinematic remapping (Handa et al., 2020) and retargeting (Rakita, 2017), which simplify the manipulation task and allow the execution of complex manipulation tasks beyond simple pick-and-place operations.

To provide operators with sufficient feedback to understand the situation and provide a suitable control interface for efficient and robust task execution, haptic feedback and model prediction (Lenz and Behnke, 2021) have been incorporated into the systems, significantly increasing work efficiency. Despite this, operators may still experience prolonged task completion times when working in close proximity to the environment, and may encounter difficulties when working remotely due to increased latency and an incomplete visual field. To address this, researchers have proposed methods for robots to complete tasks autonomously.

Techniques such as those proposed in Zhuang et al. (2019), which aid in object grasping by maximizing the contact area between the hand and the object after collision, and in Rakita et al. (2019), which construct action vocabularies to predict intent based on user actions, limited by the quantity and quality of the action vocabulary, have been proposed. Furthermore, methods such as those outlined in Rakita (2017), which relax the constraint of direct mapping between hand position and orientation and end effector configuration to smooth out the motion trajectory, have also been proposed. However, there remains a dearth of research on techniques that automatically adjust trajectories to reduce energy expenditure by operators and improve the efficiency of robot systems.

## 3 Methodology

**Figure 3:**
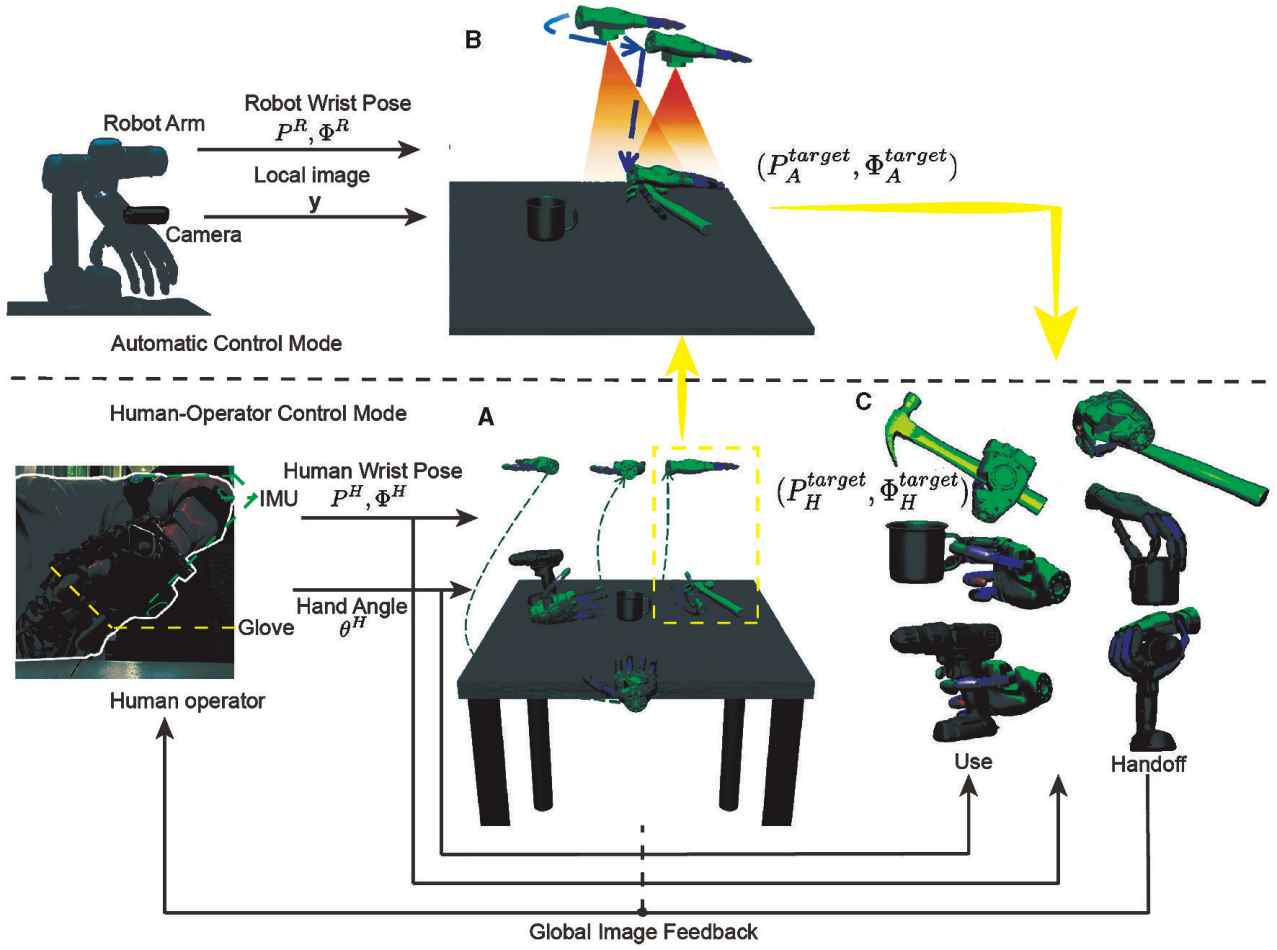
Overview of the shared control architecture for object grasping. **(A)** The operator uses the inertial measurement unit (IMU) to obtain the pose of the wrist (*P*^*H*^, Φ^*H*^) and the data gloves to obtain the position of the hand joints θ^*H*^ to select the target object. **(B)** is the Automatic Control Mode, which estimates the position and size of the object through the changes in the robot wrist pose (*P*^*R*^, Φ^*R*^) and image *y*, and then gives the robot wrist command $\left(P_{A}^{target},\Phi_{A}^{target}\right)$, where the thumb of the robot hand aligns with the edge of the object. **(C)** The human operator resumes control to choose the robot wrist pose $\left(P_{H}^{target},\Phi_{H}^{target}\right)$ based on the specific purpose of use, such as picking up or handing off the object.

The output of the robot performing the grasping task includes the wrist's pose (*P*, Φ) and hand's joint angle θ. Current control methods are categorized into automatic control, teleoperation, and shared control. Shared control is a method that combines the advantages of both automatic control and teleoperation, which synergizes human intelligence with automation to optimize task performance and efficiency. As depicted in Figure 3, the system consists of two distinctive modes: Human-Operator Control Mode and Automatic Control Mode. In Human-Operator Control Mode, the target pose of the robot wrist is $\left(P_{H}^{target},\Phi_{H}^{target}\right)$ and the hand's joint angle is $\theta_{H}^{target}$, which are controlled by the operator's wrist pose (*P*^*H*^, Φ^*H*^) and hand angle θ^*H*^. This comprehensive system harnesses the operator's cognitive capabilities to adeptly select objects and the appropriate manipulation methods, especially within complex environments. In Automatic Control Mode, the target pose of the robot wrist is $\left(P_{A}^{target},\Phi_{A}^{target}\right)$, where the thumb of the robot hand aligns with the edge of the object. The position and size of the object is estimated through the changes in the robot wrist pose (*P*^*R*^, Φ^*R*^) and image *y*. Thus, the shared control combines the two methods to achieve control of the robot $\left(P_{S}^{target},\Phi_{S}^{target},\theta_{S}^{target}\right)$.

The entire shared control process is shown in Figures 3A–C. Firstly, as shown in Figure 3A, there are three objects on the table from the YCB database: a power drill, a cup, and a hammer. The operator uses the image feedback from the global camera to select the object to be grasped and the direction of the object to be grasped, as shown by the different trajectories in the figure.The operator's finger and external joint instructions are seamlessly transmitted to the robot via a data glove and a motion capture device. Suppose that we have chosen to grasp the hammer from the top down, then as shown in Figure 3B, the automatic control mode takes over the program seamlessly. Initially, the manipulator is moved to align the object within the field of view through visual positioning, followed by a downward movement to compare pre and post movement image changes, enabling accurate depth information retrieval. This valuable data guides the manipulator to an optimized initial position for grasping. Finally, as illustrated in Figure 3C, the operator strategically considers the purpose of use and hand off, adeptly adjusting the grasping position to successfully complete the manipulation task.

### 3.1 Human-operator control mode

**Figure 4:**
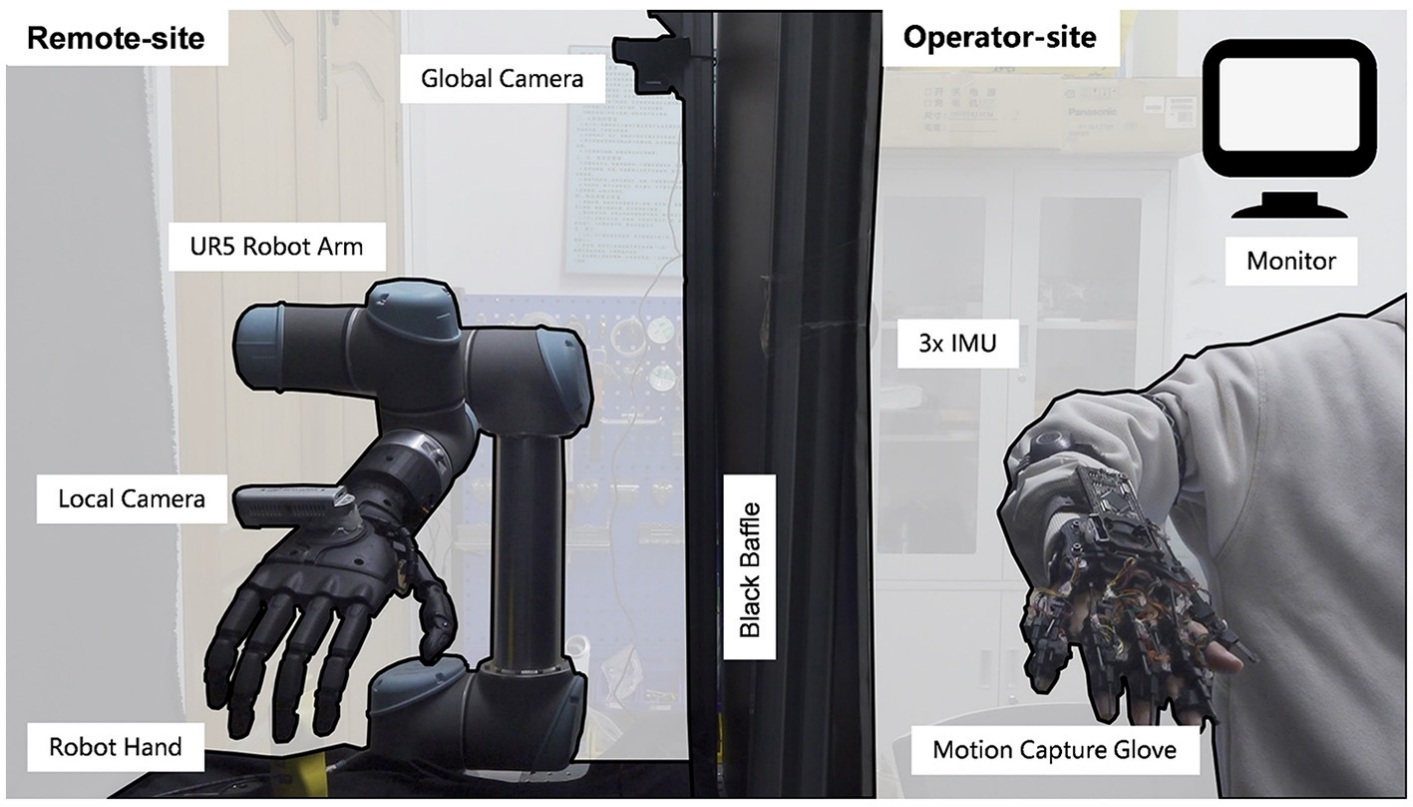
The hardware platform for shared control. The Remote-site and the operator-site are separated by a black baffle.The left half is remote-site and the right half is operator-site. The remote site consists of an UR5e robot arm and a custom robot hand to interact with the environment, a local camera to estimate the position and size of objects, and a global camera to provide feedback. The operator-site consists of three IMUs for obtaining the arm joint position and gloves for obtaining finger joint position.

In this mode, the operator leverages their cognitive abilities and physical movements to guide the robot's actions. The system implemented in this study incorporates a human-operator control mode, which facilitates selection of the target object and the manipulation method. As it is challenging to parse control signals directly from the human brain, a motion capture device was designed to interpret brain signals and transmit commands to the robot. This device includes data gloves for recording the position of finger joint and IMU for recording the position of arm joint, as the operation-site depicted in Figure 4. The data gloves use serial communication, and the IMU uses Bluetooth communication. video is available in the Supplementary material.

The shared control hardware platform comprises of an operation-site and a remote-site with a delay of approximately 30 ms. The remote site comprises a robot arm and a custom robot hand (bin Jin et al., 2022), which is optimized to replicate the natural movement of the human hand, and is equipped with 6 active and 15 passive degrees of freedom. The custom hand is designed to adapt to the shape of objects and has a loading capacity of 5 kg. Furthermore, it passed 30 out of 33 grasp tests on everyday objects according to the taxonomy of Feix et al. (2016), demonstrating its suitability for manipulation and using objects in daily life. The remote-site also includes a local camera Realsense D435 for estimating the position and size of objects, and a global camera logitech carl zeiss tessar hd 1080p for providing feedback to the operator.

In the operation site, a full-degree-of-freedom data glove is employed to record 20 finger joints with high precision (< 1 accuracy). The pip joint, which offers the largest workspace, controls the bending of the fingers. Moreover, the ab/ad joint directly corresponds to the respective human hand joints. To mitigate the jitter effect during hand movement, a low-pass filtering method is applied for a smooth and stable hand motion.

The robot hand's palm pose is entirely governed by the human hand's wrist. The control signal is derived from the wrist's pose relative to the shoulder, inferred from the angles of the arm joints recorded by three inertial sensors secured to the arm. Specifically, three IMU sensors are attached to the upper arm, forearm, and wrist to measure the angle information of the shoulder joint, elbow joint, and wrist joint. With the shoulder joint as the reference point and considering the arm's length information, the position and posture of the wrist joint relative to the shoulder are calculated for system control.

**Figure 5:**
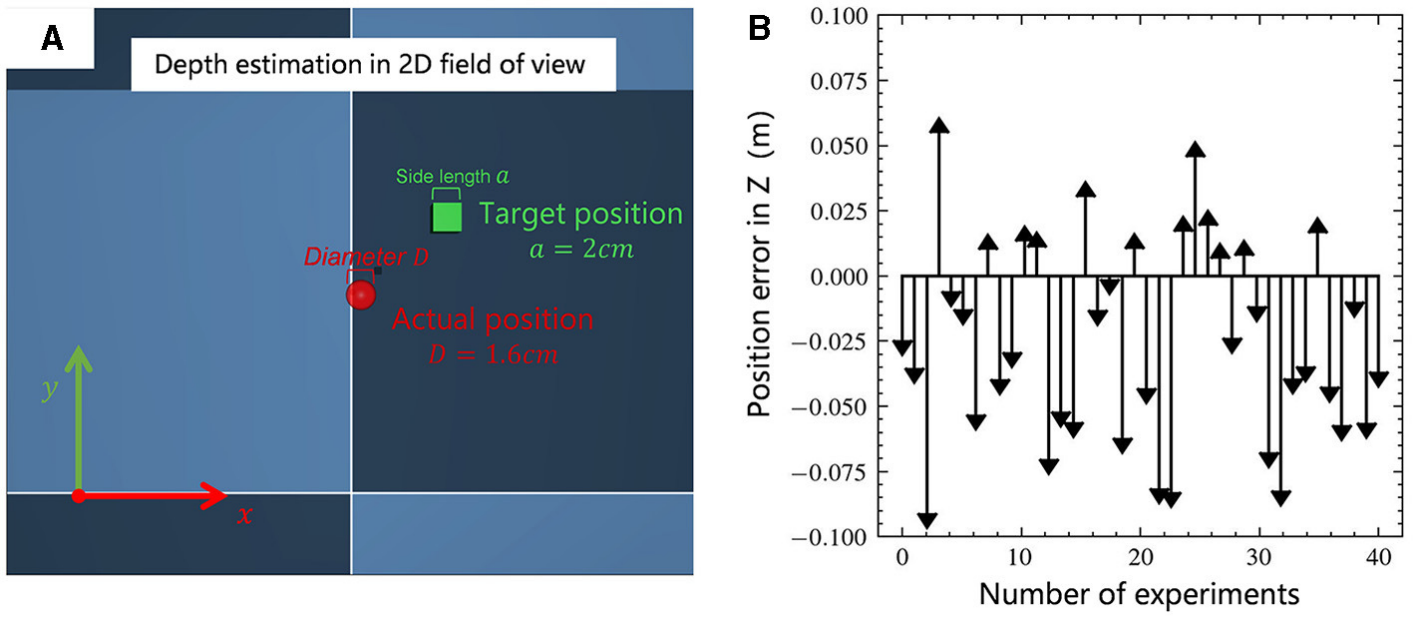
**(A)** Teleoperation of a two-dimensional visual field depth estimation scenario, with the goal of aligning the center of the red ball with the center of the green square; **(B)** Statistical results of the position error in depth direction.

In a two-dimensional visual field teleoperation system, inefficiency can significantly arise from the operator's inadequate ability to perceive depth. Figure 5, depicts the depth estimation test of the operator. Figure 5A is a test scenario in MuJoCo, where the red ball represents the actual position of the wrist, with a diameter of 1.6 cm; the green square is the target position of the wrist, with a side length of 2 cm, and each test varies randomly within the range *x, y, z*∈ [–5 cm, 5 cm]. The operator estimates the depth by comparing the size of the objects in the field of view. The camera's field of view direction is vertically downward along the z-axis, and the initial distance from the target position is 50 cm. Two groups of experiments were conducted with different operators, each experiment being conducted 20 times, resulting in the outcome shown in Figure 5B. The average error was 2.4 cm, and the fluctuation range was large. Among them, 30% of the results exceeded a range error of 5 cm, which would significantly reduce the success rate of the task.

### 3.2 Automatic control mode

The Automatic Control Mode employs the camera to verify the spatial position of the target object for grasping and subsequently initializes the position of the robot hand. Once the target object is detected within the local camera's field of view, the Automatic Control Mode is activated. The robot hand is then moved to align the camera's center with the object's center, while keeping their relative posture unchanged.

The cornerstone of this feedback control system lies in the precise measurement of the object's size and spatial measurement relative to the hand. To achieve this, a monocular vision method is employed in conjunction with the high-precision position measurement of the robot arm. This combination ensures accurate and reliable detection of the target object's location and size, enabling the system to execute manipulation tasks with efficiency and precision.

**Figure 6:**
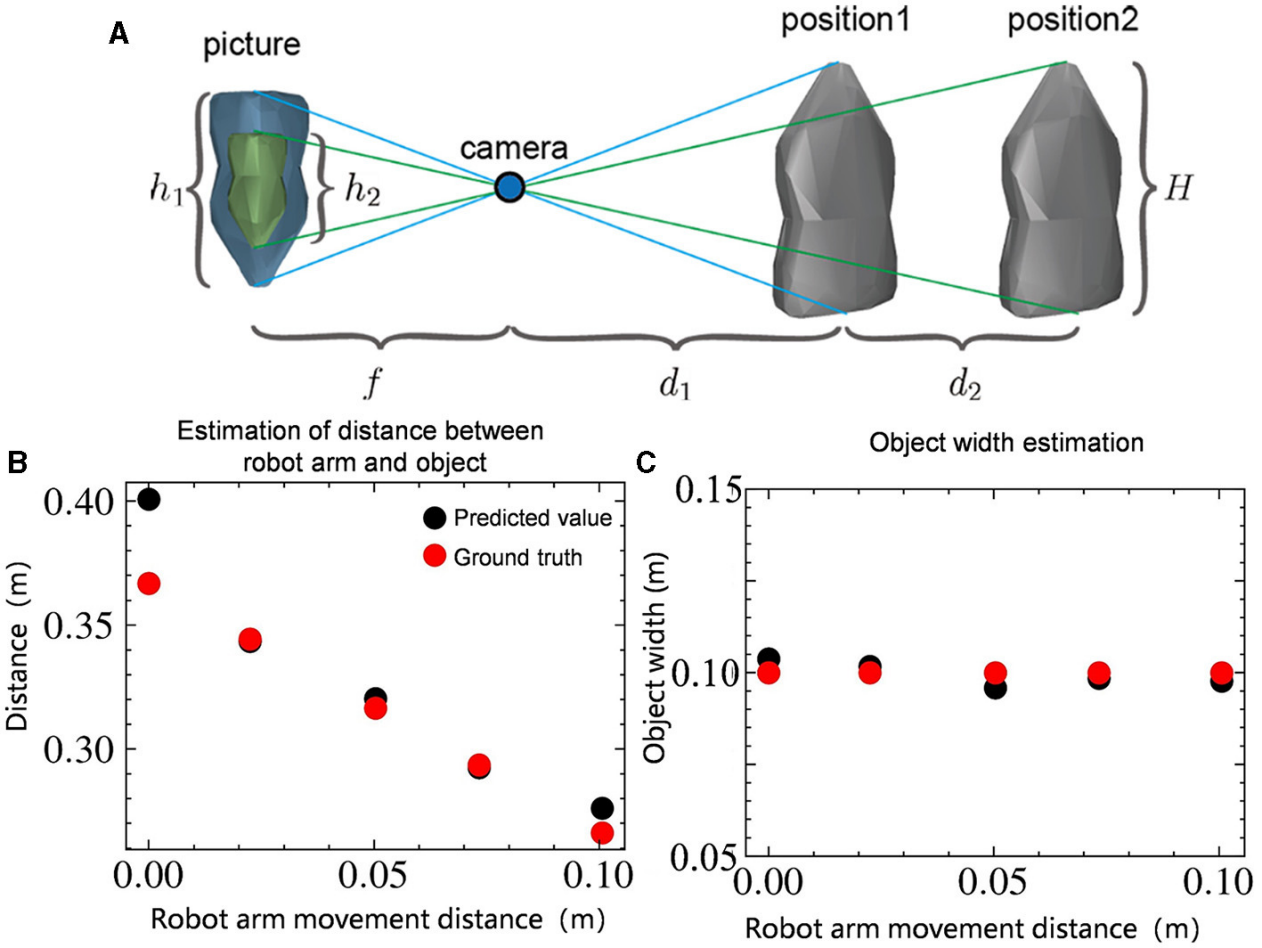
**(A)** Infer the position and size of the object based on the camera imaging principle. *H* represents the actual length of the object. *h*_1_ and *h*_2_ represent the pixel length of the object. *f* represents the focal length of the camera. *d*_1_ represents the distance of the object from the camera and *d*_2_ represents the distance moved. **(B)** Estimation of distance *d*_1_ between the size of the object and the camera. **(C)** Estimation of size of the object *H*.

In Figure 6A, the high-precision position measurement of the robot arm is used to record position differences before and after movement, along with changes in the object's image size, facilitating the inference of the object's information. Derived from the pinhole imaging principle, we can obtain the following two algebraic relationships:


(1) $$\begin{array}{l} h_{1}=\frac{f}{d_{1}}\times H \end{array}$$

(2) $$\begin{array}{l} h_{2}=\frac{f}{d_{1}+d_{2}}\times H \end{array}$$

In the above two formulas, the pixel length *h*_1_, *h*_2_ of object is known using the minimum area rectangle; The focal length of the camera *f* and the distance *d*_2_ moved by the robot arm. So we can get the real geometry length *H* of the object:


(3) $$\begin{array}{l} H=\frac{h_{1}h_{2}d_{2}}{f\left(h_{1}-h_{2}\right)} \end{array}$$

(4) $$\begin{array}{l} d_{1}=\frac{h_{2}d_{2}}{h_{1}-h_{2}} \end{array}$$

As illustrated in Figures 6B, C, this method can accurately estimate the size of the object *H* and the distance *d*_1_ between the object and the camera, reducing both depth distance estimation and object size estimation to less than 1 cm. Based on these two pieces of information, the automatic control method can achieve autonomous positioning of the robot wrist location, reducing the wrist position error in the depth direction from 2.4 cm to less than 1 cm, thus enhancing the efficiency and success rate of the task.

After successfully aligning the camera with the target object, following the aforementioned pinhole imaging principle, the robot proceeds to approach the object. Throughout this phase, the system continuously calculates the size and centroid position of the object. The robot diligently continues this approach until the distance between the hand and the object reaches a predefined threshold. At this point, the robot transitions to the target position, as illustrated in Figure 3B, where the thumb of the robot hand aligns perfectly with the edge of the object. This precise alignment facilitates subsequent operational tasks, such as grasping, manipulating, or interacting with the object effectively and accurately. Automatic control provides an effective initialization of the robot's position, thereby improving overall task performance by minimizing errors and increasing the success rate of subsequent actions.

### 3.3 Shared control framework

The shared control framework adjusts the proportional coefficient α of the above two control methods to manage the robot wrist pose $\left(P_{S}^{target},\Phi_{S}^{target}\right)$.


(5) $$\begin{array}{l} \left(P_{S}^{target},{\text{Φ}}_{S}^{target}\right)=\alpha \left(P_{A}^{target},{\text{Φ}}_{A}^{target}\right)+\left(1-\alpha \right)\left(P_{H}^{target},{\text{Φ}}_{H}^{target}\right)\\ \;\alpha =\left\{ \begin{array}{l} 1,\;\text{target object is detected }and\;|P_{A}^{target}-\\ P_{h}^{target}|<0.3\text{m}\\ 0,\;\text{otherwise} \end{array}\right. \end{array}$$

As shown in Equation 5, the proportional coefficient α is a binary variable. When the target object is within the local camera's field of view and the position commands of both control methods are within 30 cm of each other, the proportional coefficient is set to 1, utilizing the Automatic Control Mode. If a discrepancy occurs, the operator can send a position command opposite to the automatic control to increase the distance beyond 30 cm, causing the program to revert to Human-Operator Control Mode for object reselection. Additionally, when the target object is not detected or after the Automatic Control Mode has completed, the proportional coefficient α is set to 0, switching to the Human-Operator Control Mode.

**Figure 7:**
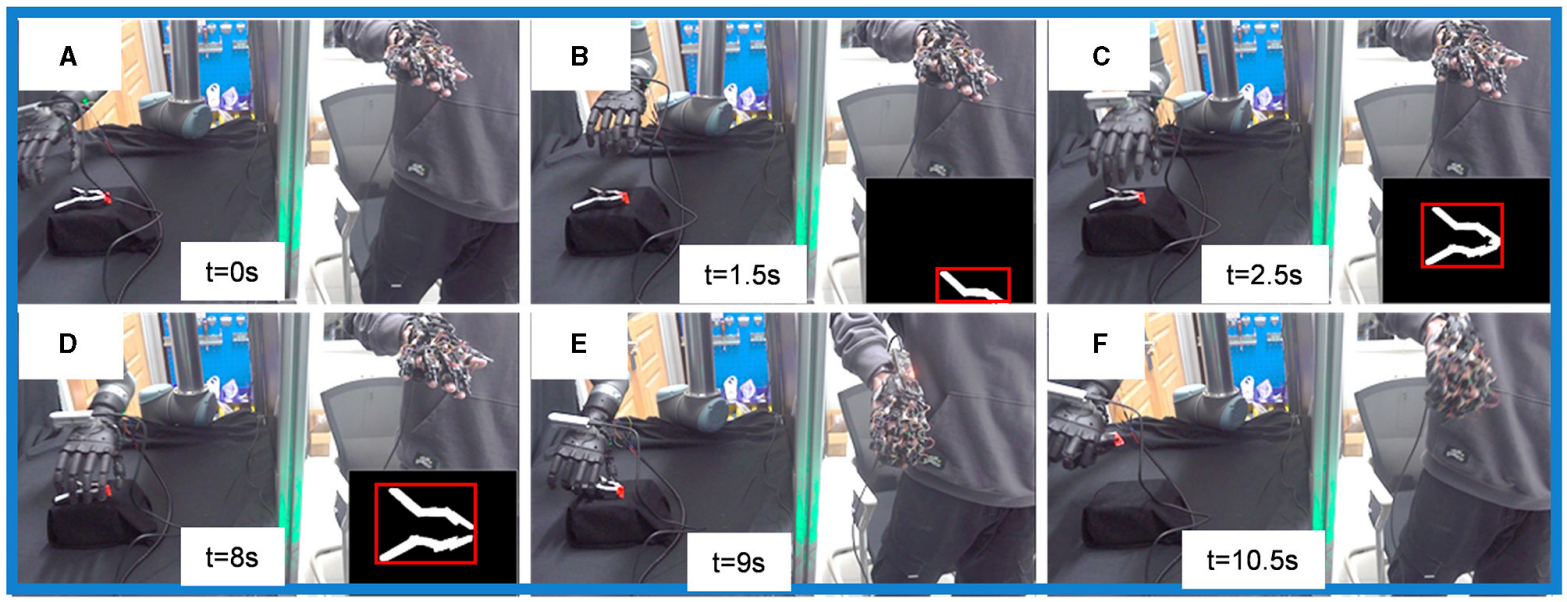
Implementation of the shared control framework in physical systems. **(A, E, F)** Are Human-Operator Mode, **(B–D)** are Automatic Control Mode.

As illustrated in Figure 7, the process of grasping an object using shared control alternates between the two control modes. Figures 7A, E, F all utilize the Human-Operator Mode. Specifically, Figure 7A represents the search for the target object, while Figures 7E, F indicate the adjustment of the wrist's pose during grasping. Figures 7B–D shows the process of the robot wrist approaching the object in the Automatic Control Mode. In the local image, object segmentation and detection are carried out using OpenCV algorithms. The process begins with Gaussian blur to reduce image noise, followed by conversion to grayscale. Otsu's thresholding method (Otsu, 1979) is then applied to segment the target object from the background. Rectangular contour detection is used to identify the object's outer contour, as indicated by the red rectangle in the figure. This allows for the calculation of the object's position on the plane to align the object's center with the camera. Finally, based on the pinhole imaging principle, two images are obtained by moving the robotic arm to a fixed position, and the estimate of object's position and size information is derived, as shown in Figures 7C, D.

Using this shared control method for object grasping significantly improves the process's efficiency. While maintaining task adaptability, the task completion time is reduced to approximately 10 seconds.

## 4 System analyze

In order to evaluate the performance of the three control methods, namely automatic, teleoperation, and shared control, a system identification method was employed to model the operation process to get quantitative metrics. Furthermore, the impact of errors and noise on the system was also analyzed. The system identification method is a widely used technique in control systems that can be used to identify the dynamic characteristics of the system by analyzing the input-output data of the system.

The results of the modeling indicate that the shared control method combines the advantages of the other two approaches and compensates for their respective limitations. Specifically, it not only exhibits fast grasping speed and smooth trajectories, similar to automatic control, but also maintains high grasping accuracy through the incorporation of human intelligence. This highlights the potential of the shared control method in improving the performance and efficiency of robot systems in real-world scenarios.

### 4.1 Modeling

Inspired by Jeka et al. (2004), velocity information is more accurate than position and acceleration. Therefore we assume that this system is a linear system and the velocity is proportional to the distance from the target position. The mathematical model of the system can be represented by the following equation:


(6) $$\begin{array}{l} x\left(t+\text{Δ}T\right)=x\left(t\right)+\left(x^{ref}\left(t\right)-x\left(t\right)\right)v\text{Δ}T+e\left(t\right) \end{array}$$

This equation can be transformed into the following form:


(7) $$\begin{array}{l} x\left(t+\text{Δ}T\right)=\left(1-v\text{Δ}T\right)x\left(t\right)+v\text{Δ}Tx^{ref}\left(t\right)+e\left(t\right) \end{array}$$

In Equation 7, the target position *x*^*ref*^(*t*) is represented by the input variable *u*(*t*) and can be considered as an input quantity of the linear stochastic dynamical system. The variable *x*(*t*) represents the current position, Δ*T* represents the time step, the scale factor *v* represents the proportionality constant between speed and distance, and *e*(*t*) represents the noise present throughout the process, which can affect the smoothness of the trajectory.

**Figure 8:**
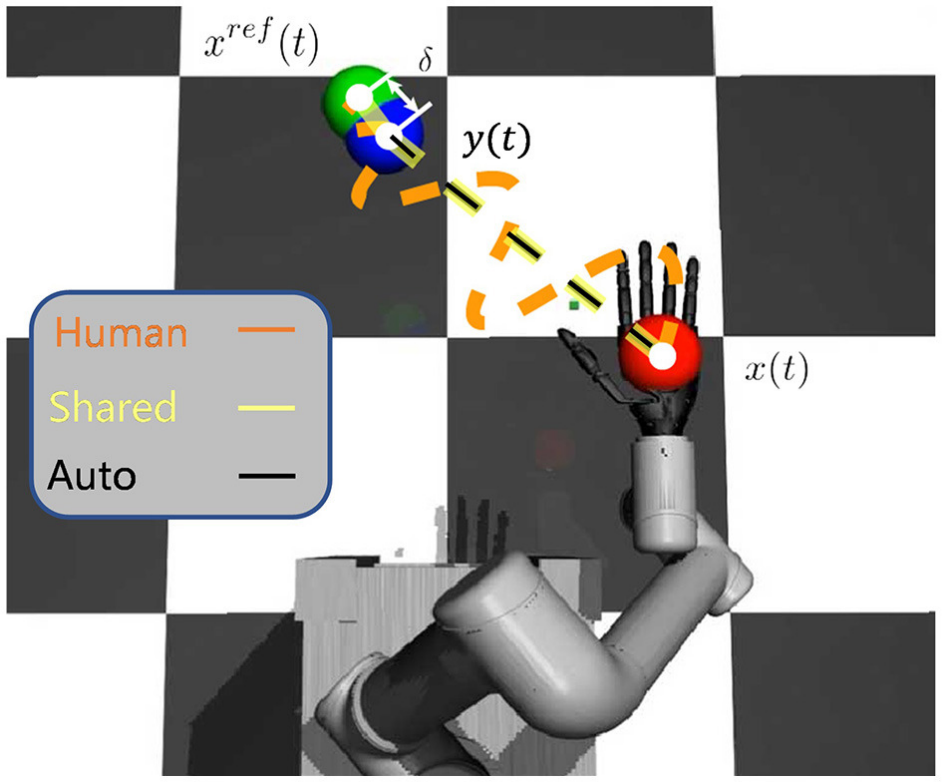
The modeling of robot system. The red ball represents current position of the robot *x*(*t*). The blue ball represents observation target position *y*(*t*). The green ball represents actual target position *x*^*ref*^(*t*). The goal of the system is to move the red ball to the green ball. Black lines, yellow lines and orange lines represent automatic control, shared control and human control respectively. The distance δ between the final position of the system and the target represents the grasping inaccuracy.

As illustrated in Figure 8, there are red, green, and blue balls in the image. The goal is to move the red ball to the position of the green ball. The red ball represents the current position *x*(*t*) of the robot hand, the green one represents the actual target position *x*^*ref*^(*t*), and the blue one represents the observation target position *y*(*t*) due to the presence of noise in the environment. The variable δ represents the grasping inaccuracy, which is the distance between the final position and the target position.

### 4.2 System identification

**Figure 9:**
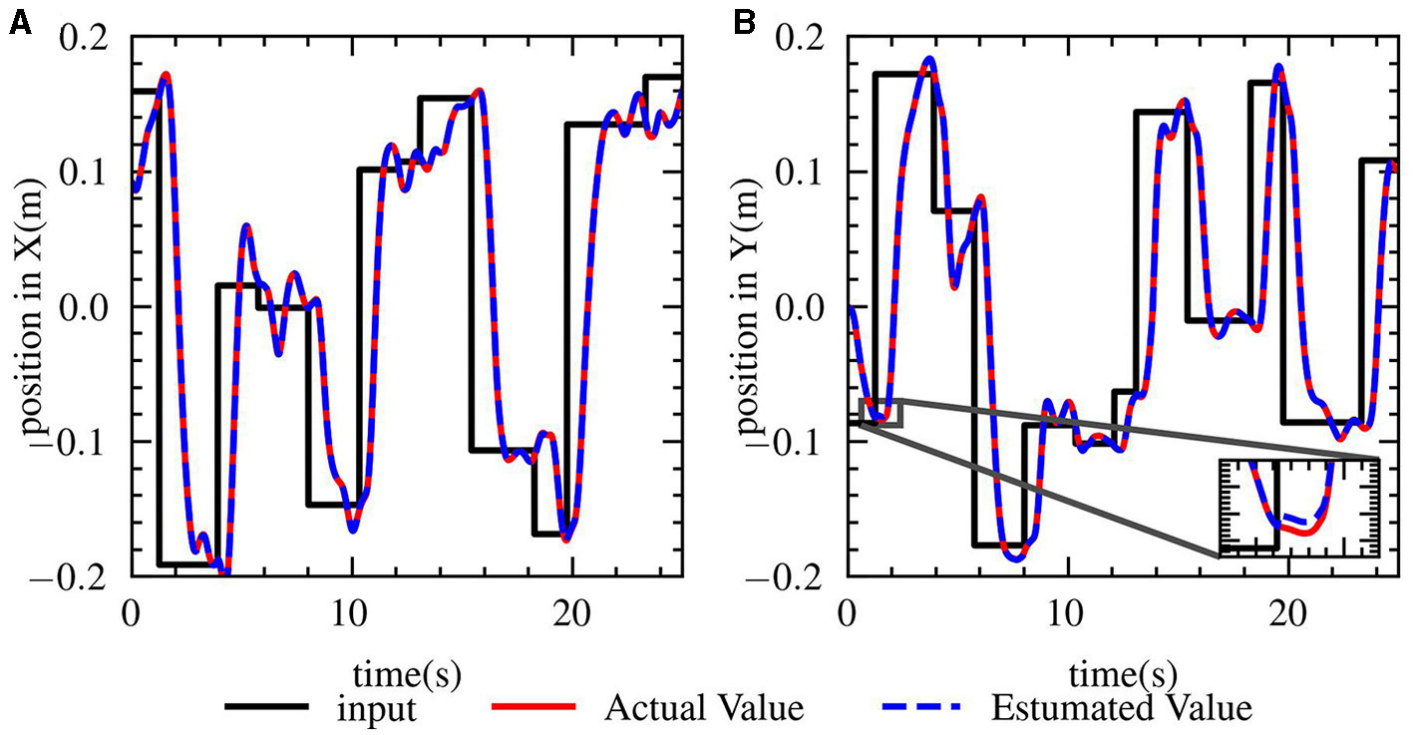
System identification for robot system. The data comes from Human Operation Mode. **(A)** Position in *x* direction. **(B)** Position in *y* direction. The black line represents the target position, the red line represents the current position of the robot and the blue line represents the position model predicted which almost coincides.

After establishing the hypothetical model presented earlier, the method of system identification was utilized to fit the model. Figure 9 illustrates that the black line represents the target position, the red line represents the position of the robot hand, and the blue line represents the data predicted by the model. As shown in lower right corner of figure, the predicted position of the model is in close agreement with the actual position, indicating that it is reasonable to describe the whole system with a linear model.


(8) $$\begin{array}{r} \begin{array}{c} \left[\begin{array}{c} x\left(t+\text{Δ}T\right)\\ y\left(t+\text{Δ}T\right) \end{array}\right]=\left[\begin{array}{cc} 1-k_{dx} & /\\ / & 1-k_{dy} \end{array}\right]\left[\begin{array}{c} x\left(t\right)\\ y\left(t\right) \end{array}\right]+\\ \;[\begin{array}{cc} k_{dx} & /\\ / & k_{dy} \end{array}]\left[\begin{array}{c} u_{x}\left(t\right)\\ u_{y}\left(t\right) \end{array}\right]+\left[\begin{array}{cc} e_{x}\left(t\right) & /\\ / & e_{y}\left(t\right) \end{array}\right] \end{array} \end{array}$$

The method of system identification was used to separately identify the system for the three control methods. The general system model is represented by Equation 8. In order to quantitatively compare the three methods, the motion trajectories and target positions under the three control methods were recorded in the scene shown in Figure 8. Three groups of experiments were conducted for each control method, and each group of experiments was repeated 20 times. The identified system parameters of the system under three control methods are presented in Table 1. The *k*_*d*_ representing speed shows that shared control is comparable to automatic control in terms of speed, and is much faster than human control. The variance of noise shows that the trajectory of shared control and automatic control is smoother than that of human control. The grasping inaccuracy δ shows that shared control and human control have higher grasping accuracy.

**Table 1:** The parameter list of the control method.

	**Autonomous**	**Shared**	**Human**
*k*_*dx*_	0.40	0.37	0.07
*k*_*dy*_	0.40	0.38	0.06
*e*_*x*_(*t*)	N(-0.47,19.1)	N(0.51,18.3)	N(-2.47,223)
*e*_*y*_(*t*)	N(-0.04,9.5)	N(0.35,23.9)	N(-4.78,297)
δ	N(8.8,41.7)	N(0.96,0.1)	N(1.09,0.09)

In summary, the method of system identification was utilized to quantitatively analyze the speed, accuracy, and trajectory smoothness of the system under different control methods. The results indicate that Shared Control is the best control method at this stage, as it combines the advantages of the other two methods to achieve fast and accurate grasping. Additionally, as previously discussed, shared control can also increase the robustness of the system, as the human operator can provide additional sensing and perception capabilities, and can intervene in case of unexpected situations.

### 4.3 Analysis of the influence of latency and noise

In the practical implementation of robot systems, noise, and latency are inevitable factors that can affect system performance. These factors can include delays caused by communications and human reaction times, as well as noise caused by sensors and the environment. Both latency and noise can degrade system performance and reduce the experience of human-robot interaction. In addition, it has been shown that increasing latency reduce accuracy and efficiency. Similarly, noise can cause errors in the measurement of the robot's position and can lead to inaccuracies in the control system. Worse yet, noise can cause the system to become unstable, making it difficult to control the robot. To evaluate the impact of these factors on the system quantitatively, simulations were conducted by incorporating various levels of delay and noise into the ideal system. The results of this analysis were then utilized to inform the design of the robot system.

Table 2 presents the parameters of the system equation under different operation conditions. Similar to Table 1, the results in Table 2 were derived from Equation 8. The data were collected from the human mode in the scenario depicted in Figure 8 by introducing different magnitudes of sensing position errors and delays. The data were obtained from three independent experimental groups, each consisting of 20 trials. The results indicate that, while small errors have minimal effect on overall system performance, large delays, greater than 100 ms, can significantly decrease both speed and accuracy. This highlights the importance of minimizing latency in the design of the robot system.

**Table 2:** Control parameters under noise and delay.

	**50 ms delay**	**100 ms delay**	**2 cm error**	**5 cm error**
*k*_*dx*_	0.06	0.03	0.07	0.06
*k*_*dy*_	0.06	0.03	0.07	0.07
*e*_*x*_(*t*)	N(-3.1,141)	N(-2.9,96)	N(-2.5,170)	N(-0.3,679)
*e*_*y*_(*t*)	N(-3.5,91)	N(-2.7,51)	N(-4.6,101)	N(-0.3,575)
δ	N(1.7,1.1)	N(3.0,3.5)	N(1.8,0.76)	N(1.6,0.6)

## 5 Results

### 5.1 System simulation

**Figure 10:**
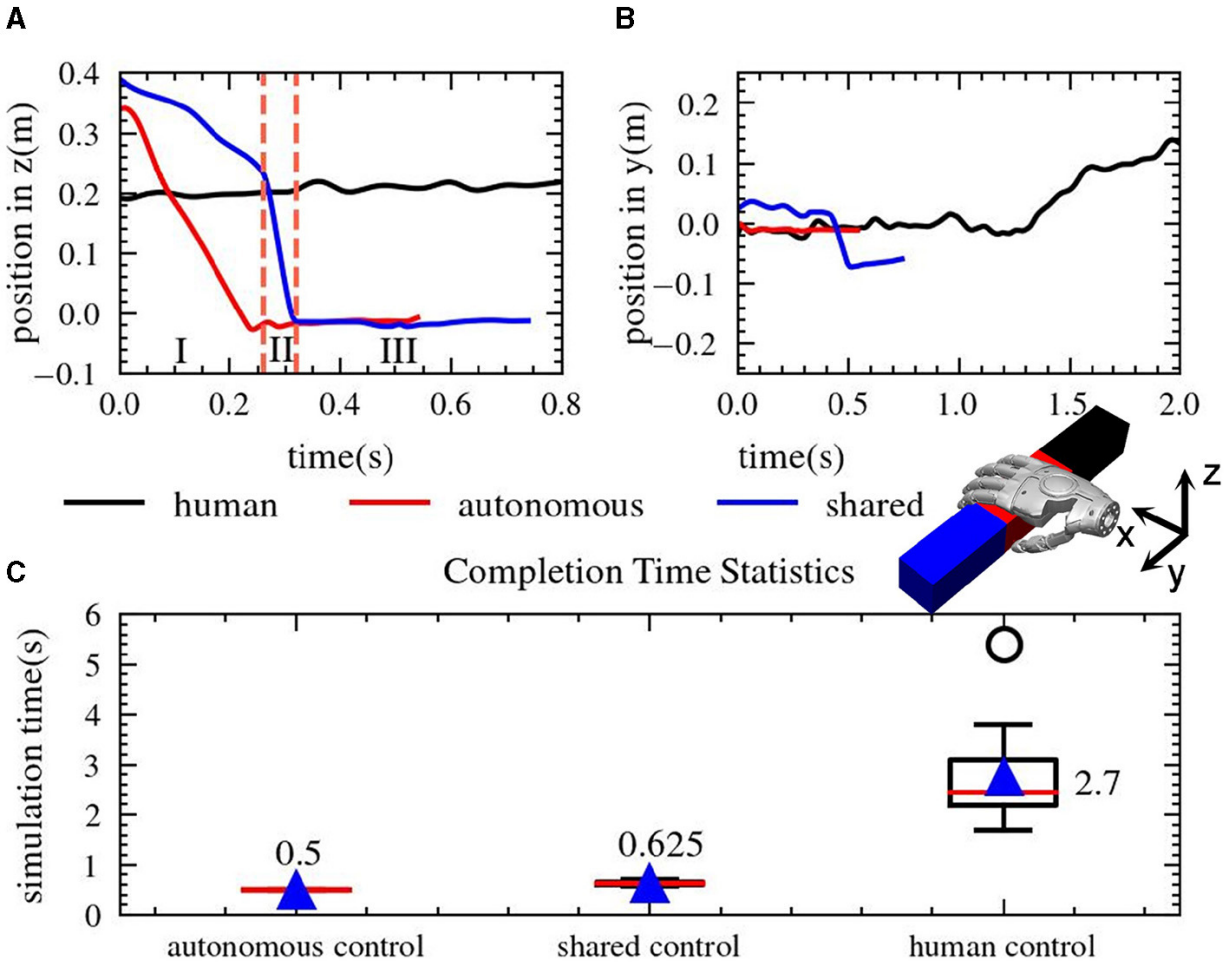
Statistics of simulation results. The figure **(A, B)** show the motion trajectories of three different control methods. **(A, B)** Shows the motion track in the z and y direction respectively. **(C)** is a box diagram of the completion time of the three control methods.

The robot system was simulated using the MuJoCo physics engine (Todorov et al., 2012). The local camera located in the palm of the robot was used to infer the position and size information of the object, providing information for the Automatic Control mode. A simple cuboid object was chosen as the test object to compare the performance of the three control methods. The statistical results of the simulations are presented in Figure 10.

In Figure 10A, the blue line representing the shared control trajectory in the *z* direction can be divided into three sections, (I) and (III) represent the manual control mode, with the target selection and gripper position adjustment implemented by the operator's instructions, (II) represents the automatic control mode implementing autonomous positioning of the wrist position. Thanks to the implementation of the automatic control mode, the shared control mode can generate smoother operation trajectories compared to the remote operation mode. Figure 10B is the trajectory on the *y* axis under three control methods, the gripper position under the automatic control mode is often constrained by the pre-set algorithm-optimized position, usually favoring the red section in the center region of the object, as shown by the red line in the plot. The remote operation mode and shared control mode, on the other hand, exhibit flexibility in the selection of positions, adjusting the grip point according to specific task requirements and real-time environmental changes. For instance, the red area on the right side of the object can be chosen under the remote control approach, while the left side of the object can be chosen for grasping using the shared control method. This flexibility in position selection gives the shared control mode greater adaptability when dealing with complex scenarios.

The task completion time under three control modes is as shown in Figure 10C, with the results being statistically represented using a box-plot. The plot mainly contains six data nodes, which from large to small represent the upper edge of the data, the upper quartile Q3, the median, the lower quartile Q1, the lower edge of the data, and the outliers represented by circles. Outliers are usually defined as values less than the first quartile (QL) minus 1.5 times the interquartile range (IQR) or more than the third quartile (QU) plus 1.5 times the IQR. The results show that the average task completion time for remote operation is approximately 2.7s, for automatic control is 0.5s, and for shared control is 0.625s. The completion time for the human operation mode is significantly higher than the automatic control mode and shared control mode, almost 5 times the time required for these two modes.

**Figure 11:**
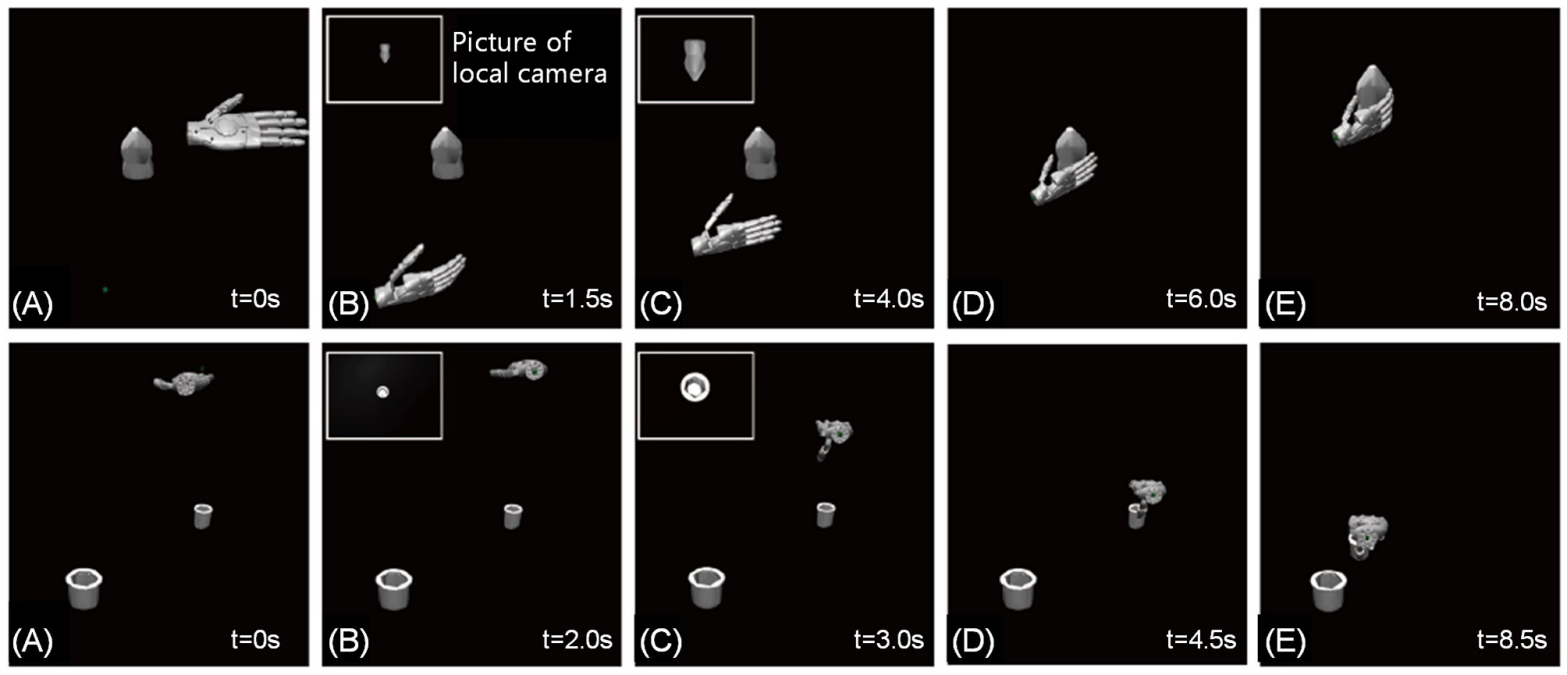
Timing diagram of shared control. **(A–E)** represents the process of grasping The upper part of the figure is grasping mustard bottle from YCB dataset from the horizontal direction. The bottom half of the figure is the cup insertion from the vertical direction.

In order to further evaluate the robustness of the proposed shared control method, simulations were conducted on irregularly shaped objects and tasks that require high precision. The objects used in the simulation were obtained from the YCB database (Calli et al., 2017). The simulation results, as shown in Figure 11, demonstrate that the shared control method is able to accomplish these tasks with ease, while traditional human-controlled methods are time-consuming and labor-intensive, and have a very low success rate. The tests encompassed acquiring object size data from both vertical and horizontal perspectives, involving precision and power grasping techniques. These results provide further evidence for the efficacy of the proposed shared control method in handling complex and challenging grasping tasks.

### 5.2 Real environment

The YCB objects were successfully grasped and used in both simulation and real-world scenarios. The results of the experiments were consistent with the conclusions drawn from the theoretical model, demonstrating the effectiveness of the shared control method. Furthermore, the performance of shared control was compared with other two. The shared control was found to be the most efficient and accurate method among the three methods.

In this study, we also evaluated the performance of a shared control framework in a simulated cup insertion task. The algorithm was successfully transferred from simulation to a real-world environment. Given the limitations of automatic control methods in adapting to various tasks, we conducted a comparison between the shared control method and human control method.

**Figure 12:**
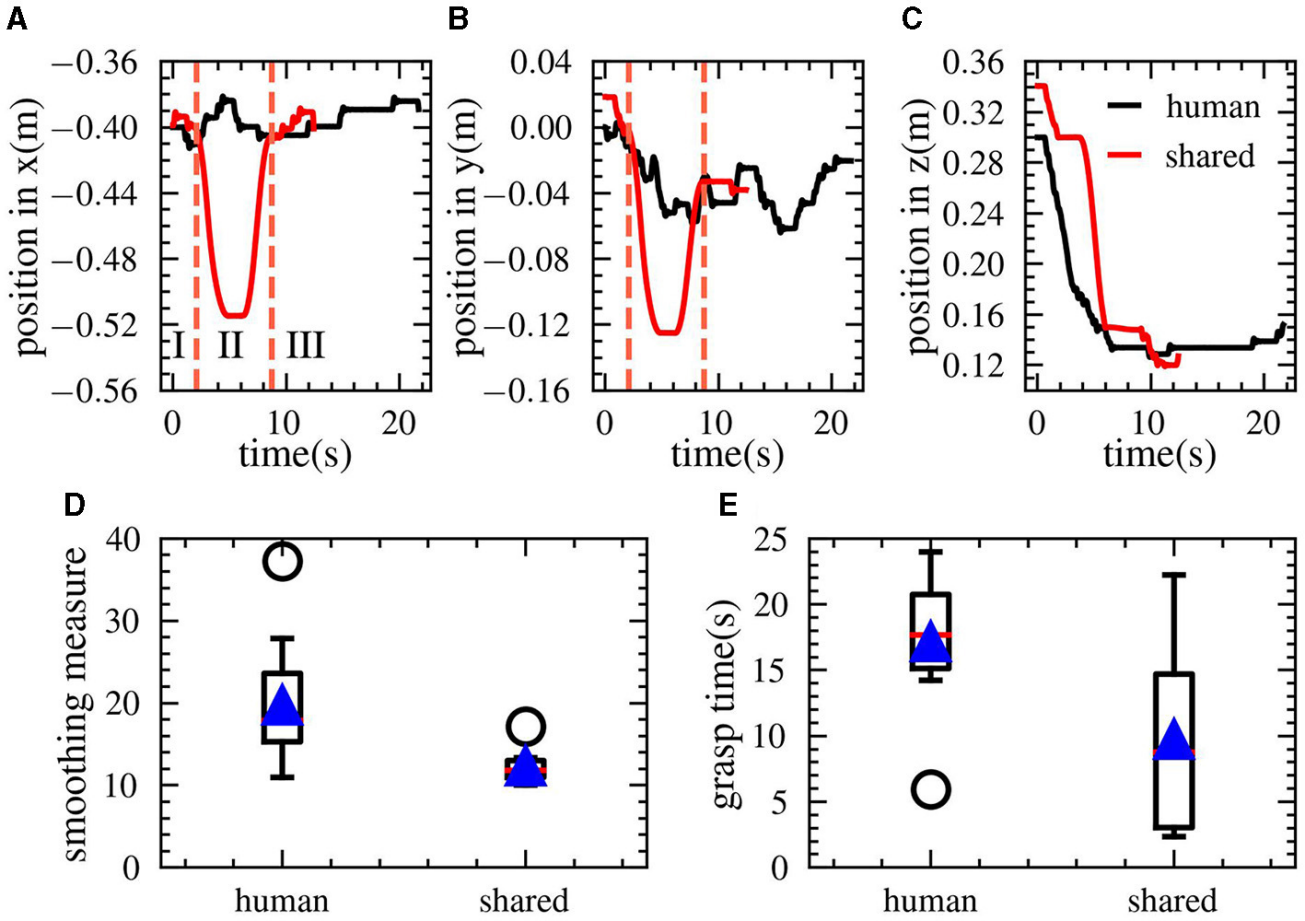
The actual experimental results of the robot stacking cups, which are part of the YCB objects. **(A–C)** Indicates the spatial position of the robot hand. *I, II*and*III* respectively represent the human mode, automatic mode and human mode in the shared control. Automatic mode will go through the two stages of first aligning the object with the camera, and then grasping the center-aligned object. Therefore, there will be two stages of moving away first and then approaching. **(D, E)** shows that shared control has faster speed and smoother trajectory than human control. The data is derived from the average results of 10 experiments.

The initial phase of the experiment involved completing tool-based tasks based on human visual perception. The results of the cup insertion task are presented in Figure 12, based on a total of 10 experiments. The Figures 12A, C depict the motion trajectories of the two methods in one experiment. Notably, the trajectory of the shared control method is smoother as evidenced by the quantitative measure of trajectory smoothness, as represented by Equation 9. Additionally, the completion time of the task was shorter for the shared control method. It should be noted that the shared control method exhibits a larger distance in both the *x* and *y* directions due to the misalignment of the camera center with the grasping center of the robot.


(9) $$\begin{array}{l} \gamma =\frac{\int_{0}^{T}|f{\left(t\right)}^{\prime \prime }{|}^{2}dt}{T} \end{array}$$

As shown in Figure 12E, compared to human operation mode, the shared control method reduced the task completion time from 20 seconds to 10 seconds, doubling the efficiency.

**Figure 13:**
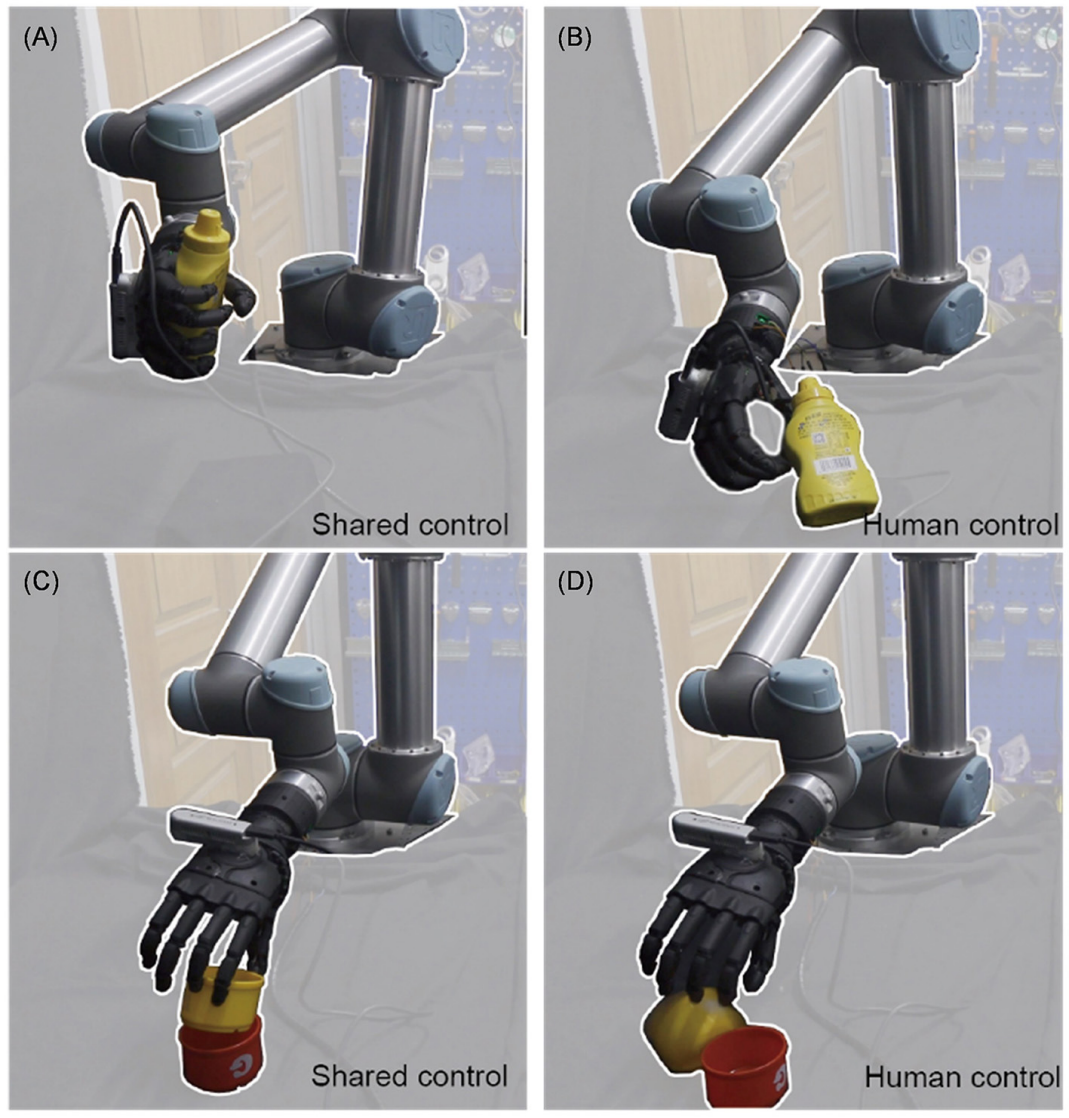
Comparison chart of shared control and human control in the real environment. **(A, C)** Representing shared control can quickly complete the task of grasping irregular objects and cup insertion. In contrast, **(B, D)** representing human control have a rather low success rate.

To further challenge the system, the difficulty of the task was increased by using a blackboard to isolate the human operator from the robot system. The operator was only able to rely on the global camera with a fixed view to operate the robot system. As depicted in Figure 13, the human control method failed to grasp the object due to the insufficient visual field, resulting in a success rate of only 40%. In contrast, the shared control method successfully completed the grasping task, demonstrating its effectiveness in overcoming the limitations posed by the restricted visual field.

The results of this study suggest that the shared control framework is an effective method for improving the performance of robot tasks. The shared control method generates smoother motion trajectories and shorter completion times compared to the human-operator control mode. Additionally, the shared control method was able to successfully complete the task even under challenging conditions where only a fixed camera view was available.

## 6 Discussion

Shared control is a control paradigm in which a human operator and an autonomous system collaborate to achieve a common goal. It has been widely studied in the field of robotics and human-robot interaction, and has been shown to be an effective approach for various tasks, such as grasping, manipulation, and navigation.

One of the key benefits of shared control is that it allows the human operator to provide high-level guidance, while the autonomous system handles low-level control. This can lead to improved performance and increased efficiency, as the human operator can focus on higher-level cognitive tasks, while the autonomous system handles the more precise and repetitive tasks. Additionally, shared control can also increase the robustness of the system, as the human operator can provide additional sensing and perception capabilities, and can intervene in case of unexpected situations.

Shared control can be implemented in various ways, such as force reflection, visual feedback, and haptic feedback. Force reflection is a method in which the human operator and the autonomous system share the control of the robot's end effector, and the robot's motion is constrained by the human operator's motion. Visual feedback is a method in which the human operator can see the robot's motion and make adjustments as needed. Haptic feedback is a method in which the human operator can feel the robot's motion, such as through a force-feedback device.

There are several challenges that need to be addressed when implementing shared control. One of the main challenges is how to design the interface between the human operator and the autonomous system. The interface should be intuitive and easy to use while also providing the necessary information for the human operator to make informed decisions. Additionally, the interaction should be seamless, with minimal delay and minimal effort required on the part of the human operator. Another challenge is how to handle uncertainty, such as unexpected situations or sensor noise. The autonomous system should be able to cope with these situations, while also providing the human operator with the necessary information to intervene, if needed.

Shared control is a promising control paradigm for robotics and human-robot interaction. It allows for improved performance and increased efficiency, while also increasing the robustness of the system. However, there are challenges that need to be addressed, such as designing the interface between the human operator and the autonomous system, and handling uncertainty. Further research is needed to address these challenges and develop more advanced shared control methods.

## 7 Conclusions

In this study, we conducted a comparison of shared control and human control methods in a simulated cup insertion task. By analyzing the errors and delays in the entire system and modeling the control methods, we were able to build a shared control robot system. We simplified the robot system into a linear system, assuming that the speed of the motion is proportional to the distance from the target position. This assumption was supported by the data generated during the movement. The shared control method combines the advantages of fast speed and smooth trajectory in the automatic control process with the advantages of human intelligence in adjusting the grasping position in the human control process, resulting in fast and accurate grasping.

The shared control robot system was successfully implemented in a physical environment, and was able to complete grasping and object manipulation tasks through image feedback. In the future, potential areas for further research include the integration of haptic feedback to enhance human presence and the use of goal prediction to anticipate which object the human operator intends to manipulate in a clustered environment.

Overall, the results of this study demonstrate the effectiveness of the shared control framework in improving the performance of robotic tasks. The shared control method exhibited smoother motion trajectories and shorter completion times compared to the human control method. Additionally, the shared control method was able to successfully complete the task even under challenging conditions where only a fixed camera view was available.

## Data Availability

The datasets generated and analyzed for this study can be found in the Github Page: https://github.com/kevincheng3/Shared-control.
