# Supplementary material for: An efficient grasping shared control architecture for unpredictable and unspecified tasks
Source: Front Neurorobot. 2024 Sep 11;18:1429952. doi: 10.3389/fnbot.2024.1429952 (PMC11422345; doi:10.3389/fnbot.2024.1429952)
Supplement: Supplementary file 1 [file Data_Sheet_1.pdf]

Supplementary Material

1 STATISTICS OF DIFFERENT CONTROL METHODS

Table S1. Teleoperation grasping data statistics

Object	Size(cm)(Calli et al., 2015)	Feature size	Second(s)
Box(Meeker and Ciocarlie, 2019)	3.5*8.9*11	7.00	29
Ball(Meeker and Ciocarlie, 2019)	14	11.28	21
Legos(Meeker and Ciocarlie, 2019)	6*3*2	3.30	29.8
Drill(Meeker and Ciocarlie, 2019)	12.5*50	9.21	30
Peg(Meeker and Ciocarlie, 2019)	2*3	2.11	61
Wire(Meeker and Ciocarlie, 2019)	5.8*5.2	5.16	19
Tape(Meeker and Ciocarlie, 2019)	9*6	7.25	30
Screwdriver(Meeker and Ciocarlie, 2019)	3.1*21.5	5.45	49
Water(Meeker and Ciocarlie, 2019)	6*18	7.98	18
Valve(Meeker and Ciocarlie, 2019)	4*4	3.69	38
Brick(Handa et al., 2020)	5*7.5*5	5.72	17.5
Spam(Handa et al., 2020)	5*9.7*8.2	7.35	22
Card sliding(Handa et al., 2020)	5.4*8.5*0.1	1.66	24
Pringles(Handa et al., 2020)	7.5*25	10.34	34
Paper cup(Handa et al., 2020)	10*15	10.56	20
Blocks (l)(Handa et al., 2020)	6.3*6.3*6.3	6.30	14
Cup insertion(Handa et al., 2020)	8.5*7.2	7.42	21.8
Tea(Handa et al., 2020)	2.5*2	2.14	42
Wallet(Handa et al., 2020)	15*8*0.008	0 .99	110
Blocks (s)(Handa et al., 2020)	2.3*2.3*2.3	2.30	38.1
Water bottle(Li et al., 2019)	6*18	7.98	23.67
Mug(Li et al., 2019)	8*8.2	7.44	18.82
Banana(Li et al., 2019)	3*19	5.12	25.8
Can(Li et al., 2019)	8.5*3.3	5.72	19.75
Apple(Li et al., 2019)	7.5	6.05	15.6
Pill(Phoenix, 2024)	1*1*2.5	1.25	30
Ball(s)(Phoenix, 2024)	4	3.22	15
Box(Phoenix, 2024)	5*9*16	8.96	13
Cup(Phoenix, 2024)	7*10	7.27	12
Spoon(Phoenix, 2024)	1.4*2*19.5	3.79	18
Knife(Phoenix, 2024)	1.4*2*21.5	3.92	14
Fork(Phoenix, 2024)	1.4*2*19.8	3.81	16
Plant(Phoenix, 2024)	25.8*24	10.79	20

For the size of the objects, a sphere is represented by the radius r, a cylinder is represented by the radius r and height$h$, and a rectangular box is represented by the side lengths a, b, c . The feature size is the cubic root of the volume.

Table S2. Automatic grasping data statistics

Object	Size(cm)(Calli et al., 2015)	Feature size	Second(s)
Box(Morgan et al., 2020)	2.6*2.6*2.6	2.60	15.5
Golf ball(Morrison et al., 2018)	4.27	3.44	15
Mug(Morrison et al., 2018)	8*8.2	7.44	15
Phillips screwdriver(Morrison et al., 2018)	3.1*21.5	5.45	15
Large marker(Morrison et al., 2018)	1.8*12.1	3.13	15
Dice(Morrison et al., 2018)	1.62*1.62*1.62	5.12	15
Banana(Morrison et al., 2018)	3*19	5.12	15
Racquetball(Morrison et al., 2018)	5.53	4.46	15
Adjustable wrench(Mahler et al., 2017)	0.5*5.5*20.5	3.82	15.5
Box(Mahler et al., 2017)	3.5*8.9*11	7.00	15.5
Cylinder(Mahler et al., 2017)	3*5	3.28	15.5
Hammer(Mahler et al., 2017)	2.4*3.2*13.5	4.70	15.5
Phillips screwdriver(Kumra et al., 2020)	2.5*2.5*2.8	2.40	20
Phillips screwdriver(Kumra et al., 2020)	2.7*2.7*3	2.58	20
Charger(Kumra et al., 2020)	7*5*3	4.72	20
Large marker(Kumra et al., 2020)	1.8*12.1	3.13	20
Truss(Kumra et al., 2020)	3*3*30	6.46	20
Tape(s)(Kumra et al., 2020)	4*2.5	3.16	20
Tape(l)(Kumra et al., 2020)	9*5	6.83	20
Chess(Kumra et al., 2020)	4*10	5.01	20
Bolt(Kumra et al., 2020)	0.8*0.8*3	1.15	20
U disk(Kumra et al., 2020)	1.5*1.5*2	1.65	20
Pen(Kumra et al., 2020)	0.9*14.5	2.10	20
Box1(Pickit3D, 2024)	50*50*100	6.30	6.7
Box2(Pickit3D, 2024)	80*50*80	6.84	6.7
Box3(Pickit3D, 2024)	30*50*120	5.65	6.7
Box4(Pickit3D, 2024)	50*35*80	5.19	6.7
Box5(Pickit3D, 2024)	60*60*60	6.00	6.7
Box6(Pickit3D, 2024)	40*80*100	6.84	6.7
Box7(Pickit3D, 2024)	30*30*60	3.78	6.7
Bottle(Pickit3D, 2024)	6*13	7.16	10
Billet(Pickit3D, 2024)	3*5	3.28	8
Cylindrical metal(Pickit3D, 2024)	1*10	1.99	7.5
Bin(Pickit3D, 2024)	3*6*8	5.24	1.7
Card(Brahmbhatt et al., 2019)	8*5*0.2	1.85	1

Table S3. Direct human hand grasping statistics

Object	Size(cm)(Calli et al., 2015)	Feature size	Second(s)
Cup(Bullock et al., 2015)	8*7	7.06	0.8
Spray bottle(Bullock et al., 2015)	15*6*3.5	6.80	2
Disk(Bullock et al., 2015)	4.5*0.6	2.12	2
Calipers(Bullock et al., 2015)	15*2.5*0.7	2.97	2
Pen(Bullock et al., 2015)	0.9*14.5	2.10	3
Paper(Bullock et al., 2015)	15*15*0.2	3.56	2.12
Plastic bottle(Bullock et al., 2015)	4*12.5	5.40	1.27
Scale(Bullock et al., 2015)	15*3.5	9.24	1
Ball(Bullock et al., 2015)	6.5	5.24	1
Chair(Bullock et al., 2015)	15*15*2	7.66	0.9
Drawer(Bullock et al., 2015)	15*8*1.5	5.65	1
Rug(Bullock et al., 2015)	15*15*1	6.08	2
Air freshener(Bullock et al., 2015)	10*4.5*4	5.65	1.72
Alarm clock(Bullock et al., 2015)	15*5	9.60	1
Bar stool(Bullock et al., 2015)	15*3	8.77	1
Basket handle(Bullock et al., 2015)	15*1*1	2.47	1
Binder clip(Bullock et al., 2015)	1.5*2.5	1.78	1.24
Book(Bullock et al., 2015)	15*15*2.75	8.52	1.5
Bottle(Bullock et al., 2015)	15*8*6	8.27	0.8
Bowl(Bullock et al., 2015)	12.5*1	4.97	0.96
Bread box(Bullock et al., 2015)	12*12*12	12.00	2.16
Brush(Bullock et al., 2015)	0.35*10	1.07	0.82
Bucket(Bullock et al., 2015)	1*15	2.28	0.78
Calculator(Bullock et al., 2015)	15*12*1	5.65	1.04
Coaster(Bullock et al., 2015)	9*0.6	3.65	0.96
Computer(Bullock et al., 2015)	15*15*0.35	4.29	0.84
Cup(Bullock et al., 2015)	7*15	8.33	0.64
Exercise equipment(Bullock et al., 2015)	5*15	10.40	0.84
Flashlight(Bullock et al., 2015)	2*15	3.92	0.68
Glass container(Bullock et al., 2015)	7.5*13.5	9.12	2
Jewelry box(Bullock et al., 2015)	15*5*3	6.08	1
Furniture(Bullock et al., 2015)	11.5*11.5*11.5	11.50	0.68
Pet bed(Bullock et al., 2015)	14*9	12.08	1
Cherry	2.5	2.02	1.5
Strawberry	4.38*5.5	4.36	1.5
Carambola	10*10*10	9.23	1.2
Apple	7.5	6.05	1.1
Avocado	9.0*10	8.60	1.1
Peach	5.9	4.76	1.2
Pear	6.62*10	7.08	1
Card	8.5*5.5*0.1	1.67	1.7
Orange	7.3	5.88	1.2
Lemon	5.4*6.8	5.38	1
Box(s)	2.6*2.6*2.6	2.60	0.6
Box(m)	4*4*4	4.00	0.7
Box(l)	6*6*6	6.00	0.65

In Table S3, parts without specific data sources are from experiments conducted by our research. Specifically, for each object, we arranged for two experimenters to conduct 10 grasping tests each, and the average of all results was taken to get the final data.

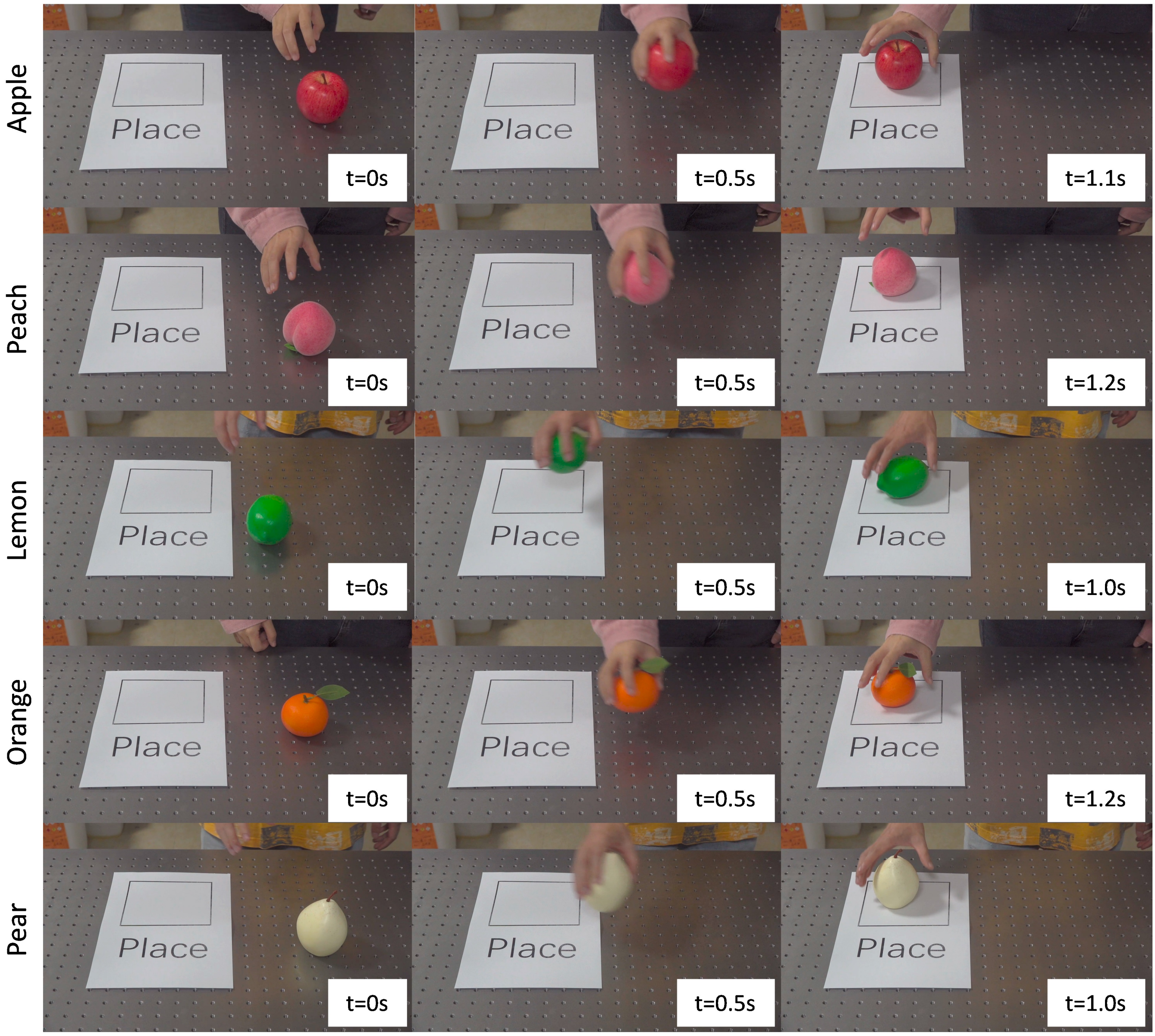

Figure S1. Direct human hand grasping experiment test

2 SEQUENCE DIAGRAM OF SHARED CONTROL GRASPING

Figure S2 realizes the shared control of autonomous wrist positioning based on the object image information.

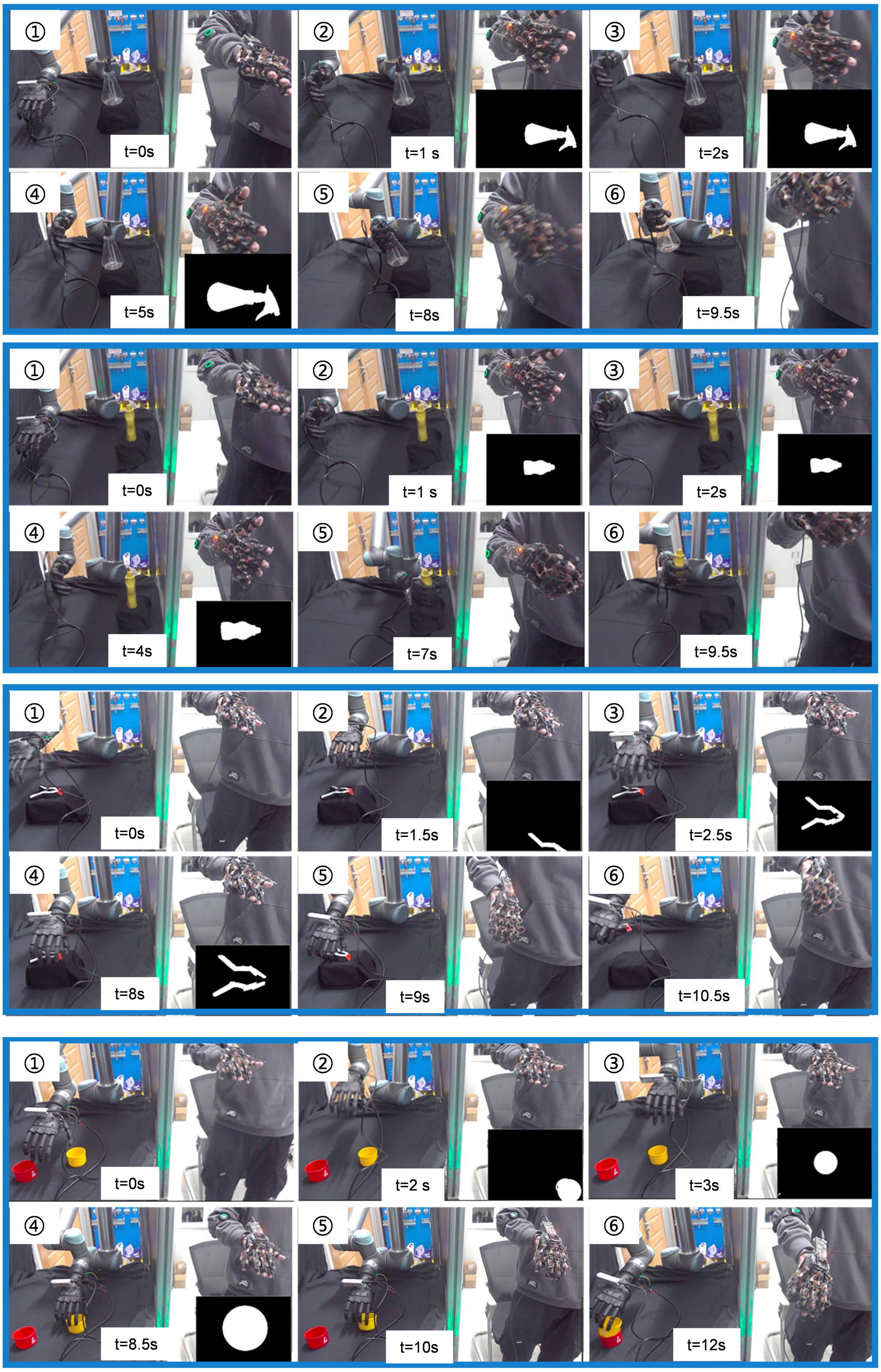

Figure S2. Sequence diagram of shared control grasping.

REFERENCES

Brahmbhatt, S., Ham, C., Kemp, C. C., and Hays, J. (2019). Contactdb: Analyzing and predicting grasp contact via thermal imaging. *Proc. IEEE Comput. Soc. Conf. Comput. Vis. Pattern Recognit.* 2019-June, 8701–8711. doi:10.1109/CVPR.2019.00891

Bullock, I. M., Feix, T., and Dollar, A. M. (2015). The Yale human grasping dataset: Grasp, object, and task data in household and machine shop environments. *Int. J. Rob. Res.* 34, 251–255. doi:10.1177/0278364914555720

Calli, B., Walsman, A., Singh, A., Srinivasa, S., Abbeel, P., and Dollar, A. M. (2015). Benchmarking in Manipulation Research: Using the Yale-CMU-Berkeley Object and Model Set. *IEEE Robotics and Automation Magazine* 22, 36–52

Handa, A., Van Wyk, K., Yang, W., Liang, J., Chao, Y. W., Wan, Q., et al. (2020). DexPilot: Vision-Based Teleoperation of Dexterous Robotic Hand-Arm System. *Proc. - IEEE Int. Conf. Robot. Autom.* , 9164–9170doi:10.1109/ICRA40945.2020.9197124

Kumra, S., Joshi, S., and Sahin, F. (2020). Antipodal robotic grasping using generative residual convolutional neural network. *IEEE Int. Conf. Intell. Robot. Syst.* , 9626–9633doi:10.1109/IROS45743.2020.9340777

Li, S., Ma, X., Liang, H., Gorner, M., Ruppel, P., Fang, B., et al. (2019). Vision-based teleoperation of shadow dexterous hand using end-to-end deep neural network. *Proc. - IEEE Int. Conf. Robot. Autom.* 2019-May, 416–422. doi:10.1109/ICRA.2019.8794277

Mahler, J., Liang, c. J., Niyaz, S., Laskey, M., Doan, R., Liu, X., et al. (2017). Dex-Net 2.0: Deep learning to plan Robust grasps with synthetic point clouds and analytic grasp metrics. *Robot. Sci. Syst.* 13

Meeker, C. and Ciocarlie, M. (2019). EMG-controlled non-anthropomorphic hand teleoperation using a continuous teleoperation subspace. *Proc. - IEEE Int. Conf. Robot. Autom.* 2019-May, 1576–1582. doi:10.1109/ICRA.2019.8794108

Morgan, A. S., Hang, K., Bircher, W. G., Alladkani, F. M., Gandhi, A., Calli, B., et al. (2020). Benchmarking Cluttered Robot Pick-And-Place Manipulation with the Box and Blocks Test. *IEEE Robot. Autom. Lett.* 5, 454–461. doi:10.1109/LRA.2019.2961053

Morrison, D., Corke, P., and Leitner, J. (2018). Closing the Loop for Robotic Grasping: A Real-time, Generative Grasp Synthesis Approach. *Robot. Sci. Syst.* doi:10.15607/RSS.2018.XIV.021

[Dataset] Phoenix (2024). Sanctuary ai

[Dataset] Pickit3D (2024). Pickit3d
